# 肿瘤相关成纤维细胞在肺癌中的研究进展

**DOI:** 10.3779/j.issn.1009-3419.2020.102.16

**Published:** 2020-04-20

**Authors:** 崇标 黄, 杰 徐, 增勋 李

**Affiliations:** 300060 天津，国家癌症临床研究中心，天津市肿瘤防治重点实验室，天津医科大学肿瘤医院加速康复外科 Department of Enhanced Recovery after Surgery, Tianjin Medical University Cancer Institute and Hospital, National Clinical Research Center for Cancer, Key Laboratory of Cancer Prevention and Therapy, Tianjin 300060, China

**Keywords:** 肿瘤相关成纤维细胞, 肺肿瘤, 肿瘤微环境, 细胞外基质, Cancer-associated fibroblasts, Lung neoplasms, Tumor microenvironment, Extracellular matrix

## Abstract

肿瘤间质在肿瘤复发和治疗抵抗中起着关键作用。肿瘤相关成纤维细胞（cancer-associated fibroblasts, CAFs）是肺癌间质中最丰富、最关键的细胞成分之一，CAFs分泌多种炎性细胞因子及细胞外基质，形成纤维增生性小生境，在肺癌发生发展的各个方面都起着促进作用。肺癌CAFs具有多种不同的起源，主要由正常肺成纤维细胞在受到肿瘤源性细胞因子作用后所转化而来。不同CAFs亚群具有较大的异质性，其功能及作用机制也具有很大差异性；这给靶向CAFs的临床转化应用带来了很大的挑战。本综述重点阐述了CAFs的特性和功能研究中的新进展，同时强调CAFs在肺癌发生、发展中起到的作用及其特异性。

## Abstract (en)

Tumor stroma plays key roles in promoting tumor recurrence and treatment resistance. Cancer-associated fibroblasts (CAFs) are one of the most abundant and key components in the stroma of lung cancer. CAFs secrete a variety of inflammatory cytokines and extracellular matrix to form a desmoplastic tumor niche, which play important roles in the occurrence and development of lung cancer. CAFs are mainly derived from normal lung fibroblasts, which are transformed by tumor-derived cytokines. The diverse sources of CAFs lead to great heterogeneity in different CAFs subgroups. Although many studies support that CAFs promote tumor growth, but evolving data also argue for their antitumor actions. The putative bimodal function in oncogenesis of CAFs bring great challenges to the clinical application of CAFs-targeted therapies. This review focuses on the characteristics and functional research of CAFs, and emphasizes the roles and specificity of CAFs in the development of lung cancer.

目前肿瘤的发病率在迅速增加，其死亡率占人类死亡原因的第2位；其中肺癌的发病率及死亡率均高居首位，已超过癌症死因的20%^[1]^。近年来随着研究的深入，对肿瘤发生、发展机制的认识已经取得相当大的进展，研发了很多新型有效的抗肿瘤药物，例如靶向药物、程序性死亡受体1（programmed cell death protein 1, PD-1）免疫药物以及抗肿瘤血管生成药物等，这些药物在某些肺癌患者治疗中展现出较好的抗肿瘤效果^[2]^。然而，由于肿瘤异质性以及复杂的肿瘤微环境，大部分肺癌患者治疗后会出现耐药、复发及转移，导致治疗失败，致使肺癌的死亡率仍居高不下。

肿瘤微环境是一个极其复杂的内环境系统，由肿瘤间质细胞、细胞代谢产物以及各种理化因素如pH值、乏氧、间质液体压力等组成，密切参与肿瘤发生、生长、转移及耐药中的每一个进程^[3]^。肺癌肿瘤微环境中重要的间质细胞包括肿瘤相关成纤维细胞（cancer-associated fibroblasts, CAFs）、肿瘤相关巨噬细胞、免疫细胞、血管内皮细胞以及定向分化的间叶细胞等^[4]^。CAFs是一种梭形细胞，是肿瘤微环境中最丰富的基质细胞之一。大量研究表明，CAFs在肿瘤发病机制中具有重要作用，具有重要的临床意义。CAFs通过建立并重塑细胞外基质（extracellular matrix, ECM）结构，使肿瘤细胞易于侵入血管；CAFs通过分泌多种生长因子、细胞因子和趋化因子，与肿瘤细胞或其他基质细胞相互作用，促进肿瘤的进展^[5]^。由于CAFs固有的可塑性，使其相对容易分离和培养，因而得到了广泛的关注。在过去的数十年里，CAFs被认为是开发新型抗癌药物的重要靶标。然而，由于CAFs具有高度异质性并且缺乏特定的标记物，对于CAFs的起源、分型和生物学功能的认识还不够全面，仍存在很多争议。目前关于CAFs的研究大多是基于细胞水平或小鼠模型，以CAFs为直接靶点的临床试验还较少，还没有成功的临床转化性研究。深入解析CAFs在肿瘤发展中的功能和分子机制，是开发靶向CAFs治疗策略的先决条件。本综述阐述了目前对CAFs的特征、生物学和异质性的研究进展，并讨论了CAFs在肺癌发生、发展、转移和治疗中的作用。

## 1 CAFs的特征及起源

正常组织中的成纤维细胞来自于ECM内的静息间充质细胞。它们可以在伤口愈合、组织炎症和纤维化期间被激活，以促进修复和再生。癌症又被称为“永不愈合的伤口”，肿瘤ECM内的成纤维细胞激活后可以促进肿瘤炎症和肿瘤纤维化^[6]^，因此又被称为CAFs。与静息的成纤维细胞相比，CAFs形态通常更大，细胞核凹陷，胞质具有更多的分支结构^[7]^。另外，激活的CAFs增殖和迁移能力更强^[8]^。正常组织中的成纤维细胞代谢和转录活性非常低，而CAFs的代谢却很活跃。CAFs最独特的特征是具有很强的促ECM合成功能^[8]^。CAFs还可以产生多种生长因子和促炎性细胞因子，特别是转化生长因子β（transforming growth factor-β, TGF-β）、血管内皮生长因子（vascular endothelial growth factor, VEGF）、白细胞介素6和CXC趋化因子12（C-X-C motif chemokine ligand 12, CXCL12），促进肿瘤血管生成，向肿瘤微环境中招募免疫抑制细胞诱发肿瘤免疫逃避^[9]^。

越来越多的研究^[6]^表明CAFs是一个异质的细胞群，而这种异质性可能是由于CAFs的起源不同而造成的。类似于伤口愈合相关成纤维细胞，CAFs可以从正常的组织内募集并激活成纤维细胞^[10]^，其激活很大程度上依赖于肿瘤微环境的刺激，如局部缺氧、氧化应激、邻近肿瘤细胞和浸润免疫细胞释放的生长因子等。TGF-β、表皮生长因子（epidermal growth factor, EGF）、血小板衍生生长因子（platelet derived growth factor, PDGF）和成纤维细胞生长因子2（fibroblast growth factor 2, FGF2）是成纤维细胞募集和活化的关键调节因子^[11]^。此外，免疫细胞源性白细胞介素-1β通过激活成纤维细胞中的核因子-κB，促进成纤维细胞的生长并促其分泌炎性因子^[12]^。除局部来源外，部分CAFs可由非成纤维细胞（如上皮细胞、血管、脂肪细胞、周细胞和平滑肌细胞）转化而来^[13, 14]^。研究发现上皮细胞和内皮细胞分别经历上皮-间质转化和内皮-间质转化，表达成纤维细胞特异性蛋白-1（S100 calcium binding protein A4, S100A4），诱导出成纤维细胞功能及表型^[15]^。CAFs也可来源于肺癌、胶质瘤、乳腺癌、胃癌和胰腺癌中典型的骨髓间充质干细胞^[16, 17]^。与癌细胞相比，CAFs的基因组突变较少，通常被认为更具有遗传稳定性；然而最新的研究^[18]^表明，成纤维细胞向CAFs的不可逆转化可能是由表观遗传改变所驱动的。总的来说，CAFs的起源还没有完全阐明。

## 2 CAFs在肺癌中的异质性

肺脏是一个复杂的器官，其组织内含有多种间质细胞，成纤维细胞是维持肺脏正常生理功能的最重要间质细胞之一。成纤维细胞的异质性是其具有不同表型和功能的关键原因^[19]^。基于全基因组表达谱测序的研究发现，源自肺脏不同部位的成纤维细胞间存在显著差异性，按照其异质性可分成不同成纤维细胞亚群^[20, 21]^。Pechkovsky等^[22]^学者从人肺实质、近端支气管和远端薄壁组织等不同解剖区域分离出成纤维细胞，进行基因组测序，发现源自肺实质的成纤维细胞自发地发育出α-平滑肌肌动蛋白（alpha-smooth muscle actin, α-SMA）高表达的肌成纤维细胞表型，这些细胞是分泌ECM、引起蛋白沉积的主要细胞群体，可能在特发性肺纤维化、纤维增生性疾病以及肺癌的发生和发展中起到关键作用。Tsukui等^[23]^学者通过构建博来霉素诱导肺纤维化的体内模型来研究成纤维细胞群的基因表达谱，他们发现肺成纤维细胞中骨桥蛋白（osteopontin, OPN）高度过表达，可作为CAFs活化的标志物。有趣的是，OPN在衰老的成纤维细胞中表达水平显着增加，并且被认为是衰老基质促肿瘤进展的关键介质之一^[24]^。

细胞谱系追踪已经被广泛用来识别和表征成纤维细胞亚群的起源^[25]^，但在鉴定小鼠肺成纤维细胞的起源上，单细胞转录分析方法更为常用。目前，研究者已经在小鼠健康肺和纤维化肺中分别鉴定出5个成纤维细胞亚群，这些细胞亚群包括肌成纤维细胞（Acta2^+^）、Col3a1基质成纤维细胞（Col3a1^+^, Itga8^+^）、Col4a1基质成纤维细胞（Col4a1^+^, Dcn^+^）、脂肪成纤维细胞（Lp1^+^）和间充质祖细胞（CD52^+^）；而在纤维化的肺中鉴定出了6个成纤维细胞亚群，除了上述的5个亚群外，还发现了一种高表达PDGF受体β的成纤维细胞类型^[26]^。在非小细胞肺癌（non-small cell lung cancer, NSCLC）的间质中，PDGF受体α的高表达与较好的预后呈正相关。然而，PDGF受体的表达水平对NSCLC患者预后的影响是不确定的^[27]^。因此，PDGF是否能作为NSCLC治疗靶点要区别看待，需要进一步研究。在最近的一项研究中，Hoshino等^[28]^学者发现在肺癌细胞A549的异种移植模型中，血管外膜成纤维细胞在肺血管周围环境形成了一个促肺癌进展的特殊小生态，这些细胞比非血管来源的肺成纤维细胞具有更高的促肿瘤活性。其原因可能与这些成纤维细胞高表达平足蛋白（podoplanin, PDPN）有关，而很多研究表明平足蛋白具有促进肿瘤发生和淋巴结转移的作用。不同的CAFs亚群中成纤维细胞活化蛋白（fibroblast activation protein, FAP）的表达水平也不相同。Kilvaer等^[29]^用免疫组化方法检测人NSCLC组织切片，发现αSMA和FAP在不同的CAFs上表达水平不一致，FAP在肌纤维母细胞型CAFs上表达水平较低。这项研究^[30]^还指出了用FAP抗体标记肿瘤微环境中CAFs的特异性不高，因为FAP在巨噬细胞中也有表达。由于FAP表达的非特异性，以FAP为靶点的免疫治疗可能引起明显的治疗副作用，例如肌肉中FAP表达阳性可引起恶病质。一项关于NSCLC患者的研究^[31]^发现，CAFs表达的谷氨酰胺-果糖-6-磷酸转氨酶2是肿瘤微环境中葡萄糖摄取和代谢重编程的原因，可能是导致CAFs的异质性及肿瘤微环境复杂性的核心机制之一。

## 3 CAFs对肺癌生物学行为的影响

### 3.1 CAFs促肿瘤发生

与正常成纤维细胞不同，CAFs在促进非致瘤性上皮细胞的生长和恶性转化中起主要作用。该现象首先在人前列腺癌小鼠模型中被发现。研究者将转导SV40的永生化前列腺上皮细胞与CAFs共同移植到小鼠体内，结果发现CAFs可以促使永生化人前列腺上皮细胞的肿瘤发生和进展^[32]^。与静息状态的成纤维细胞相比，活化的成纤维细胞或CAFs与肿瘤细胞相互作用更能促进肿瘤的发生和肿瘤生长^[33]^。Navab等^[34]^学者发现整合素α11β1在CAFs中过表达，是分泌纤维胶原的基质细胞的特异性受体，具有调节癌基质的硬度、促进NSCLC的致瘤性和肿瘤转移的生物学作用。Zhu等^[35]^学者发现整合素α11可通过调节成纤维细胞中胰岛素样生长因子-2的表达，显著地促进NSCLC肺癌细胞的致瘤性。丝裂原活化蛋白激酶（mitogen-activated protein kinase, MAPK）通路被认为是介导肺癌发生发展的关键信号，其作用是在基质相互作用中提供早期的促肿瘤发生信号。Brichkina等^[36]^学者发现，在受到促瘤信号刺激后，MAPK/p38通路激活，驱动成纤维细胞合成特异性的透明质酸，并活化成为CAFs，促使肺癌发生及增殖。总之，上述研究表明CAFs具有调节癌症基质硬度并促进NSCLC的致瘤性和转移潜能，在肺癌的肿瘤形成中起着重要作用。

### 3.2 CAFs对肿瘤的抑制作用

尽管CAFs具有很强的促肿瘤作用，但一些CAFs亚群具有明确的抑瘤作用，这进一步支持了肿瘤微环境中CAFs异质性的概念。据报道，PDPN表达阳性的CAFs是小细胞肺癌（small cell lung cancer, SCLC）患者术后的有利预后因子。与对照组相比，过表达PDPN的CAFs能明显抑制SCLC细胞的增殖速度；而通过shRNA抑制CAFs的PDPN表达后，SCLC细胞增殖速度明显上升。在手术切除SCLC标本中，PDPN表达阳性CAFs的SCLC患者的细胞周期相关因子Geminin阳性肿瘤细胞含量显着高于PDPN阴性CAFs的患者。因此，表达PDPN的CAFs具有抑制SCLC细胞的生长的作用^[37]^。Mishra等^[38]^学者采用四维肺癌模型，体外证实肺CAFs可以显著抑制肺癌的转移能力。Chen等^[39]^学者研究发现，CAFs也可以抑制肺癌的发生过程。他们研究了肺鳞癌细胞从增生、发育不良到获得侵袭性的病理生理过程。该研究发现干细胞转录因子（SRY-box transcription factor 2, SOX2）的过表达足以介导肺癌细胞球状体中从增生到发育不良的转变；而CAFs的存在又能抑制SOX2的功能，使细胞团重新形成腺泡样结构，从而抑制肿瘤细胞的侵袭性。该研究结果证实CAFs对肺癌的发生具有抑制作用。因此，寻找更为特异、可靠的细胞表面标志物，以区分促肿瘤和抗肿瘤的CAFs亚群，是开展CAFs靶向治疗的前提。

### 3.3 CAFs促肿瘤血管生成

肿瘤的生长需要新生血管来提供氧气和营养^[40]^，而CAFs具有促进肿瘤血管生成以满足恶性肿瘤的生长需求的作用。CAFs通过分泌基质细胞衍生因子1（stromal derived factor 1, SDF-1）募集内皮祖细胞促进体内新血管形成^[41]^。作为促血管生成因子的主要来源，CAFs可产生丰富的VEGFA、PDGFC、FGF2、OPN和分泌型卷曲相关蛋白2，以刺激或加剧肿瘤组织的血管生成重编程^[42]^。另外，CAFs还可以通过产生一系列ECM控制的生化和生物物理变化来调节肿瘤基质内的硬度、弹性和间质液压，间接调节血管生成和肿瘤内血流^[43]^。

### 3.4 CAFs促肿瘤转移

肿瘤的转移是一个多阶段过程，癌细胞侵袭、迁移，破坏基底膜，进入周围血管，随着血流播散并种植于远处组织，形成转移灶。研究^[44]^表明，S100A4缺陷小鼠缺乏成纤维细胞分化，其体内的肿瘤不能发生转移，表明S100A4^+^ CAFs细胞在促进转移扩散中具有明确的作用。肿瘤微环境中的趋化因子有助于肿瘤转移。王立民等^[45]^学者报道，CAFs通过分泌白细胞介素6激活肺癌细胞Janus激酶2/信号转导与转录激活子3信号通路，促进NSCLC细胞表达上皮-间质转化标志物E-钙粘蛋白和波形蛋白，并诱导其发生上皮-间质转化、上调转移相关基因基质金属蛋白酶2和VEGF的表达，从而明显地增强肺癌细胞的转移潜能。NSCLC组织和原代CAFs细胞中高表达白细胞介素-22。CAFs可通过白细胞介素-22激活了NSCLC细胞的PI3K/Akt/mTOR通路，显著地促进了肿瘤细胞的迁移和侵袭能力^[46]^。此外，S100A4表达阳性的组织驻留型成纤维细胞可形成促转移小生境，在促血管生成和抗凋亡中起着关键作用^[47]^。总体而言，成纤维细胞在远隔器官中产生转移前小生境，并引发随后的转移事件，因此又被称为转移相关的成纤维细胞（metastasis-associated fibroblasts, MAFs）。MAFs是否是从原发肿瘤被募集到转移部位还是由转移部位的组织驻留型成纤维细胞激活而来目前还未完全阐明，需要进一步研究来确定。

### 3.5 CAFs促肿瘤耐药

关于肿瘤微环境诱导肺癌耐药的研究也越来越多。宋尔卫教授团队^[48]^在乳腺癌和肺癌中鉴定出CD10^+^ GPR77^+^ CAFs亚型，该CAF细胞通过为肿瘤干细胞提供小生境来促进肿瘤形成和化疗耐药。另外，研究^[49]^发现CAFs促进A549细胞产生胰岛素样生长因子-2，可通过作为ABC转运体P-GP的诱导物来介导耐药。据报道，由癌细胞和CAFs产生的蛋白多糖-甘氨酸通过CD44作用于癌细胞，可诱导肿瘤细胞Nanog表达，使之产生抗药性^[50]^。另一项研究^[51]^发现，顺铂治疗后的肺腺癌内的CAFs通过上调白细胞介素-11的表达，激活STAT3抗凋亡途径赋予肺癌细胞耐药性。研究者还发现白细胞介素-11受体表达水平高的患者对顺铂的反应较差。

### 3.6 CAFs与肿瘤免疫

CAFs可以通过招募免疫抑制性细胞到肿瘤局部，诱导肿瘤免疫抑制作用。研究^[52]^发现肺癌组织内的CAFs可通过分泌CXCL1、CXCL2、CXCL5及CCL3等趋化因子，招募粒细胞样髓源性抑制细胞到肿瘤内，形成免疫抑制性肿瘤微环境。另一方面，CAFs促进了调节性T细胞的募集、分化和存活，有助于免疫抑制微环境的形成和维持^[53]^。CAFs还涉及通过多种机制影响各种免疫细胞对免疫抑制表型的功能，例如，CAFs通过分泌CCL2、白细胞介素-6、粒细胞-巨噬细胞集落刺激因子及肿瘤坏死因子α等细胞因子促使单核细胞向M2型肿瘤巨噬细胞转化，抑制肿瘤免疫反应^[54]^。CAFs除了介导肿瘤微环境中免疫细胞的募集和功能分化之外，多项研究^[55, 56]^发现CAFs还可通过影响免疫检查点分子PD-1、T细胞免疫球蛋白粘蛋白-3、细胞毒性T淋巴细胞相关蛋白4和淋巴细胞活化基因-3等的表达，直接抑制细胞毒性T淋巴细胞的杀伤功能。一项近期的研究发现肺腺癌CAFs可以通过抗原呈递作用，直接与活化的CD8^+^ T细胞相互作用，通过PD-L2和FAS配体接合诱导T细胞死亡。此外，当受到抗原负载的CAFs调节时，抗原特异性T细胞对肿瘤细胞的杀伤能力明显受损，表明CAFs能够驱动肿瘤特异性T细胞的功能障碍和死亡，导致肿瘤细胞存活能力增强^[57]^。

## 4 靶向CAFs治疗

CAFs在癌症发展过程中发挥的促肿瘤功能使其成为有潜力的抗肿瘤治疗靶标。然而，目前针对CAFs开展靶向治疗面临着许多障碍和挑战。由于目前缺乏CAFs细胞表面特异性标志物，因此难以在不损伤正常组织情况下精确靶向CAFs。然而，随着我们对CAFs生物学认识的日益深入，对CAFs靶向治疗的热情正在增加，目前已经报道了许多临床前研究。FAP最早被作为CAFs的靶标，在超过90%的上皮来源肿瘤的CAFs中均有表达。研究^[58, 59]^发现敲除FAP表达能抑制肿瘤生长并显著增强抗癌药物的肿瘤组织摄取。在一项肺癌Ⅰ期研究中，对FAP阳性晚期肺癌患者使用FAP抗体，该抗体能聚集到肿瘤部位并产生特异性结合，但遗憾的是临床上没有观察到明显的应答反应^[60]^。然而，在另一项关于肺癌、胰腺癌异种移植模型研究^[61]^中，给予一种与美登木素生物碱结合的新型抗FAP单克隆抗体FAP5-DM1，可以长期抑制肿瘤的生长，甚至完全抑制肿瘤从而引起肿瘤消退。很多研究发现TGF-β是介导CAFs与肿瘤实质细胞相互作用的一个重要细胞因子，可促使肿瘤发生侵袭和转移。虽然在过去十余年中很多临床前研究测试了大量抗TGF-β抗体和TGF-β受体Ⅰ激酶的抗肿瘤作用，以探索靶向TGF-β的有效性，但是这些研究结果都不甚理想，因此靶向TGF-β信号通路的有效性仍然存疑，需要进一步验证^[62]^。

上述研究所采用的靶点都不是CAFs特异性表达的分子，这极大地阻碍了靶向CAFs的精准性，因此寻找CAFs特异性靶点并深入研究作为靶点的可行性可能更具有意义。CD10和GPR77是肺癌、乳腺癌等CAFs的新型成纤维细胞表达的特异性膜蛋白^[48]^。研究^[48]^发现用特异性抗体阻断GPR77能显著降低了CD10^+^ GPR77^+^ CAFs的浸润和ALDH1^+^肿瘤干细胞的比例，抑制异种移植模型中肿瘤的生长并增强化疗敏感性。

另一种策略是靶向CAFs的来源细胞。前面提到，MDSC细胞是CAFs的重要前体细胞之一。研究^[63-65]^发现，多种药物可以通过不同机制抑制MDSC，从而阻断CAFs的促肿瘤作用。肿瘤可以通过分泌CCL2、CSF-1及G-CSF等多种趋化因子，招募MDSC进入肿瘤组织。阻断这些趋化途径后，能阻断MDSC进入肺脏，从而抑制肿瘤血管生成、肿瘤生长及肿瘤转移。全反式维甲酸可以诱导MDSC向树突细胞（CD11c^+^ I Ab^+^），巨噬细胞（F4/80^+^）和粒细胞（Gr1^+^ CD11b^-^）的分化，并改善CD4^+^ T细胞应答^[66]^。一项肺癌的临床研究^[67]^发现全反式维甲酸可以增加了DC疫苗功效，降低外周血中MDSC数量并改善抗原特异性CD8^+^ T细胞的免疫应答。

虽然理论上靶向CAFs等细胞基质可以作为一个抗肿瘤治疗途径，但迄今为止还没有抗基质疗法成功地转化为临床实践。其中可能的原因是这些基质是组织内重要的组分，在维持组织稳态中起到关键作用，去除CAFs等基质成分可能反而会促进肿瘤进展^[68]^。因此，将促肿瘤型ECM重编程为抑制肿瘤微环境似乎更为可行。另外，单独靶向细胞基质或许是不够的，同时靶向肿瘤实质细胞和基质细胞可能是一种更有效的策略。

## 5 总结

基质在肿瘤的发生、生长、侵袭和存活等多个病生理过程中起到关键作用。深入理解肿瘤基质与实质细胞的相互作用及机制是全面揭示肿瘤分子机制的重要组成部分。随着研究的深入，肿瘤基质已经成为抗肿瘤治疗的重要靶标之一。CAFs是肺癌基质内的重要细胞成分，在肺癌发生和发展中发挥着关键作用，因此CAFs是靶向肿瘤基质治疗策略的最有潜力靶点。然而，目前靶向CAFs的治疗还存在很多困难，例如缺乏特异性细胞表面标志物。为了成功建立针对CAFs的新型靶向治疗，需要进行高质量的基础研究和转化研究，以进一步揭示CAFs、癌细胞和其他间质细胞之间的相互作用及调控机制。
